# Radio-Immunotherapy: A Case Report of ‘Abscopal Hyper-Progression’?

**DOI:** 10.7759/cureus.10117

**Published:** 2020-08-29

**Authors:** Ioannis M Koukourakis, Vassilis Kouloulias, Michael I Koukourakis

**Affiliations:** 1 2nd Department of Radiology, Radiotherapy Unit, Attikon University Hospital, National and Kapodistrian University of Athens, Medical School, Athens, GRC; 2 Radiotherapy-Oncology, Medical School, Democritus University of Thrace, Alexandroupolis, GRC

**Keywords:** pd-1, radio-vaccination, hyper-progression, radiotherapy, immunotherapy

## Abstract

The radio-immunization effects of radiotherapy with abscopal tumor regressions have been documented in several experimental and clinical studies. Here, we present a patient with bladder cancer and relapsed metastatic disease to the left supraclavicular/axillary area and left lung. Concurrent weekly hypofractionated radiotherapy of both areas (8Gy/fraction/week, four fractions in total) and bi-weekly immunotherapy with anti-programmed cell death protein 1 (anti-PD-1) monoclonal antibodies resulted in complete regression of the axillary metastatic masses and of the lung metastasis, three months after the onset of therapy. In CT scans, however, a sternum infiltrating mass growing proximal to the margins of the radiotherapy fields was strikingly evident, while multiple hepatic metastases also appeared. Lymphopenia during radio-immunotherapy was recorded. The current report does not confirm the abscopal effects of radio-immunotherapy and furthermore, suggests that progressive disease or even hyper-progression may occur in a subgroup of patients. Although radio-vaccination is a well-established phenomenon, it is evident that we still miss major aspects of host/tumor-immune interactions with radiation.

## Introduction

The radio-immunization effects of radiotherapy have been documented in several experimental and clinical studies. The irradiated cancer cell can become a vaccine by enhancing antigen presentation to dendritic cells, up-regulating the interferon (IFN) type-I response, overexpressing surface antigens, and secreting chemo-attracting chemokines [1]. This radiation-induced vaccination, further to the enhancement of local tumor control, may also prime T-cells to attack metastasis residing away from the radiotherapy fields, producing the so-called ‘abscopal effects’ of irradiation (‘ab’: away from, ‘scopus’: target).

Several clinical studies have reported remission of metastatic disease during radiotherapy of the primary or other metastatic sites [2,3]. Experimental studies also show that radiotherapy, especially when combined with immune checkpoint inhibitors, produces strong positive abscopal effects with shrinkage of tumors outside the radiotherapy fields [4-6]. Nevertheless, such a phenomenon is strikingly rare in clinical practice of radiotherapy when given without immunotherapy. Metastasis often appears weeks or months after treatment, strongly questioning the existence of abscopal effects. It has been postulated that the low dose per fraction (2Gy) delivered during conventional radiotherapy has poor radio-vaccination effects. Indeed, doses per fraction around 8Gy are demanded to induce Interferon Type-I response by cancer cells [7].

In the current study, we report an unusual case, where radio-immunotherapy produced a differential effect between in-field gross disease and out-field subclinical disease, bringing forward the existence of a fragile immunological balance between abscopal effects and hyper-progression.

## Case presentation

A 78-year old man was admitted in February 2018, with a high-grade urothelial carcinoma of the bladder, staged by CT-scan/MRI radiological examination as T3-N0-M0. The patient developed progressive disease infiltrating throughout the bladder wall while he was under Bacillus Calmette-Guerin (BCG) intra-vesical therapy for superficial bladder carcinoma. He was, subsequently, treated with cisplatin radio-chemotherapy. A CT-scan and cystoscopy performed two and a half months after the completion of therapy confirmed complete response.

**Figure 1:**
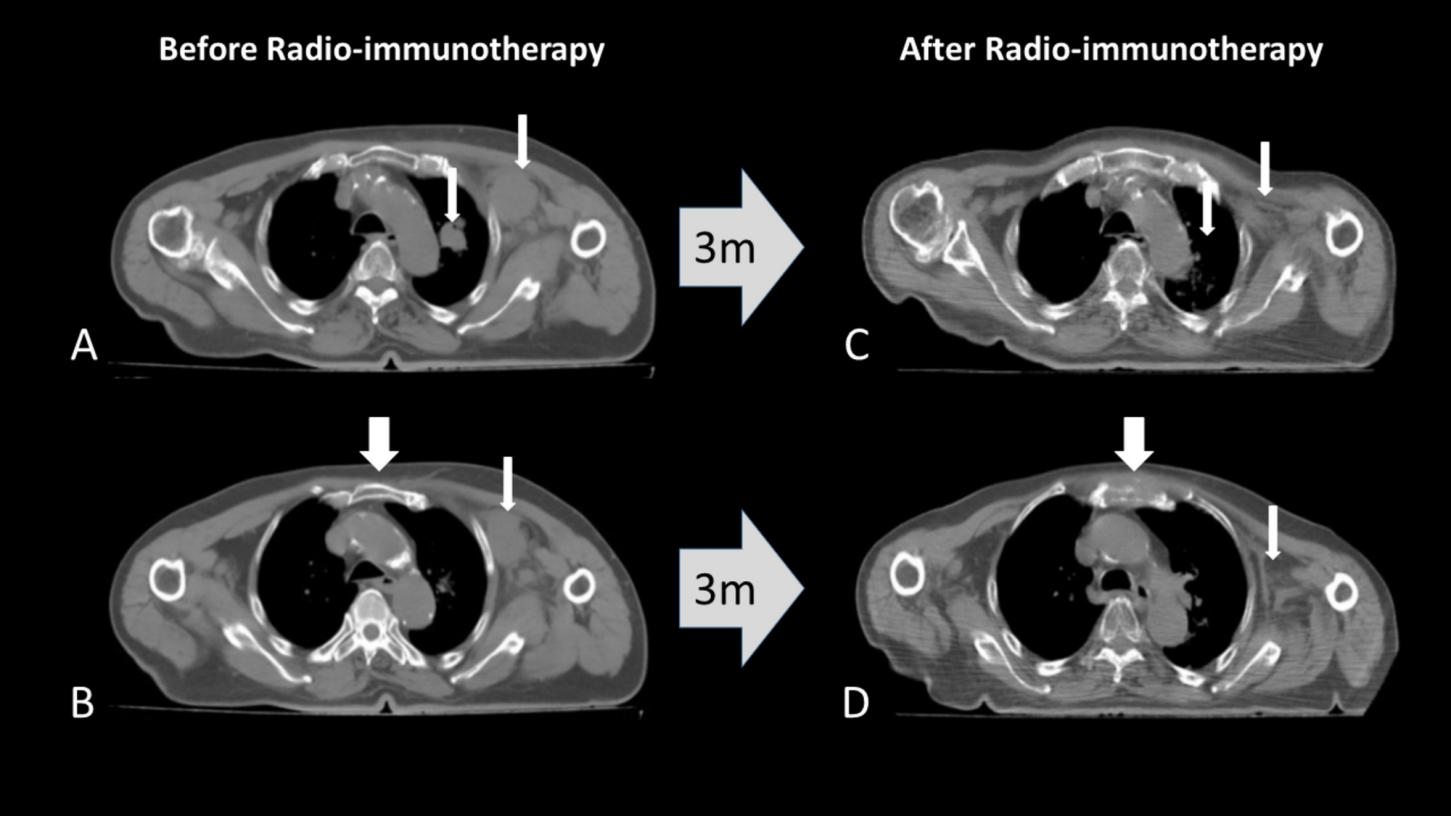
A metastatic bladder carcinoma to the left axillary/sub-clavicular area and upper left lung lobe (A, B: thin arrows). Complete response of the disease is noted three months after the onset of radio-immunotherapy (8Gy/week for four fractions) with anti-PD-1 monoclonal antibodies (C, D: thin arrows). Progression of disease immediately outside the radiation portals is evident, with a newly appearing rapidly growing tumor mass infiltrating the sternum (B, D: wide arrows). m: months; anti-PD-1, anti-programmed cell death protein 1

In August 2019, the patient was admitted for his regular follow-up, reporting persistent back pain. Cystoscopy was normal, and CT-scan showed no evidence of metastatic splanchnic disease but was suspicious for bone metastasis. Bone scintigraphy confirmed bone metastasis to the lumbar L3/L4 and thoracic T12 vertebra. As chemotherapy has a poor overall activity on bone metastatic disease, it was decided to treat the patient with radiotherapy alone, applying a wait and see policy. The patient received five consecutive fractions of 5Gy radiotherapy, directed to the T11-L5 vertebra, achieving a complete remission of the pain. In November 2019, a new CT-scan revealed a large sub-clavicular/axillary mass and nodes, as well as a metastatic tumor mass of 3cm to the left lung upper lobe (Figure 1A, 1B). There was no recurrent bladder disease or clinical progression of bone disease. The disease was considered as a progression of the known metastatic bladder carcinoma and we did not perform any additional biopsy of the nodal mass.

 

**Figure 2:**
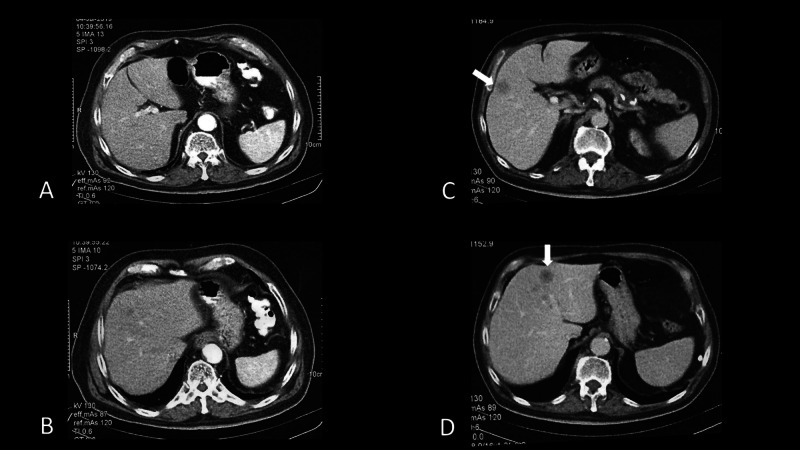
Liver metastases evident in the abdominal CT-scan examination at the time of documentation of sternal mass progression (C, D), a finding that was undetectable at CT-scans before the onset of immunotherapy (A, B).

The patient was recruited in a radio-immunotherapy study approved by the local Ethics and Scientific Committee (ES7/30-7-19 - DS15/13-11-2019). For bladder cancer, anti-PD-L1 immunotherapy has been approved regardless of the expression status of PD-L1 [8]. Therefore, immunohistochemical detection of PD-L1 was not performed. The relatively poor performance status and the advanced age also favored the decision to offer immunotherapy rather than chemotherapy. The patient gave written informed consent. The patient received immunotherapy with anti-programmed cell death protein 1 monoclonal antibody (anti-PD-1; nivolumab) (240mg every two weeks) and concurrent hypofractionated radiotherapy directed to the supra-/sub-clavicular and axillary area, as well as to the lung metastasis, using a volumetric modulated arch therapy (VMAT) technique. 8Gy per fraction was delivered weekly for three fractions, while a fourth fraction of 8Gy was given to the remnant disease following a new simulation and re-planning. In February 2020, a new CT-scan was performed to assess response, the patient having accomplished six cycles of immunotherapy. CT-scan showed complete disappearance of the irradiated tumor masses (Figure 1C, 1D). However, a new rapidly growing mass (reaching 4cm in diameter within 3 months, from a previously undetectable state) infiltrating the sternum was recorded immediately next to the lateral inner borders of the irradiation fields (Figure 1B, 1D). Moreover, two gross liver metastases (around 2cm in diameter) were evident in the abdominal CT-scan examination, a finding that was undetectable at CT-scans before the onset of immunotherapy (Figure 2). Immunotherapy was interrupted, and the patient started chemotherapy and radiotherapy to the sternal mass. The patient did not suffer from baseline lymphopenia (1520/μl), while a drop of lymphocyte counts was noted during therapy (600-1200/μl).

## Discussion

Our clinical experience on the combination of radiotherapy with immune checkpoint inhibitors predicts, with certainty, a forthcoming change in the oncology clinical practice. Striking response rates obtained and prolonged survival of otherwise untreatable patients is readily achieved in the vast majority of patients. However, abscopal effects induced by radiotherapy are difficult to document. This is because regression of metastatic disease outside the radiation portals may well occur as a result of the immunotherapy itself, without any contribution from a putative radio-vaccination.

Unexpected rapid disease progression in patients under immunotherapy, the so-called hyper-progression, is a well-documented phenomenon occurring in about 10-20% of patients [8]. This phenomenon also explains the strange finding of randomized trials, where the progression-free interval during the first six months of treatment is worse in the group of patients treated with immunotherapy, compared to one of the patients receiving chemotherapy [9,10]. It has been suggested that for the diagnosis of hyper-progression, documentation of a tumor growth rate of at least double to the one recorded before the onset of immunotherapy is demanded [11].

An on-going clinical trial in our department examines the role of a radio-vaccination-inducing hypofractionated radiotherapy schedule of 8Gy per fraction, delivered to recurrent and metastatic disease sites, together with anti-PD-1 immunotherapy. The current case report presents a bladder cancer patient who developed the metastatic disease to the supraclavicular/axillary area and lungs. Concurrent weekly hypofractionated radiotherapy and bi-weekly immunotherapy resulted in complete regression of the metastatic masses, three months after the onset of therapy. In CT-scans, however, a sternum infiltrating tumor mass, growing at the margins of the radiotherapy fields, and multiple hepatic metastases appeared. These findings may suggest a hyper-progression of the disease outside the radiation portals, as these overgrew from the subclinical level to gross tumor masses within three months, under immunotherapy. Although the exact definition of hyper-progression in our protocol is along with the generally accepted rule of tumor growth rate of at least double to the one recorded before the onset of immunotherapy, this cannot be applied in the current report as there was no detectable tumor before the onset of treatment. Rapid progression of the disease due to lack of response to immunotherapy may also explain the findings, but a clear cut-off between hyper-progression and rapid growth is unclear. In-field disease, however, showed an equally rapid and striking tumor regression, eventually attributed to enhanced immunogenic death within the irradiated tumor, which could indicate that at least the irradiated tumor was responsive to immunotherapy.

Although radiotherapy induces radio-vaccination by directly acting on cancer cells and tumor stroma, it also has an intense immunosuppressive systemic activity, easily detectable as lymphopenia [12]. In the current report, lymphocyte counts were reduced by up to 60% during radio-immunotherapy. This effect is mainly caused by radiotherapy as, in our experience, patients receiving anti-PD1 therapy do not suffer from lymphopenia. Aside from lymphopenia, radiotherapy may repress specific cytotoxic and promote regulatory T-cell lymphocytic subpopulations, a side effect documented by previous studies from our group [13,14]. Radiotherapy, therefore, is a double-edged sword as on the one hand, it promotes the recognition of cancer cells by lymphocytes, but on the other hand, it suppresses their cytotoxic potential. Whether baseline or therapy-induced immuno-suppression is the cause of the abrogation of abscopal effects of radiotherapy or of hyper-progression emerges as a plausible hypothesis.

## Conclusions

Radio-vaccination is a well-established phenomenon in both preclinical and clinical studies. This effect is a prerequisite for the manifestation of the abscopal effects of radiotherapy, which, however, are seldom noticed in the clinical practice, presumably because of the immunosuppressive activity of radiation on cytotoxic cells. The current report does not confirm the abscopal effects of radio-immunotherapy and furthermore, suggests that hyper-progression may occur in a subgroup of patients, unveiling the naivety of our scientific knowledge that fails to understand the complexity of the interactions between radiation and immunity.
